# Supplementary material for: Predicting visual function by interpreting a neuronal wiring diagram
Source: Nature. 2024 Oct 2;634(8032):113–23. doi: 10.1038/s41586-024-07953-5 (PMC11446822; doi:10.1038/s41586-024-07953-5)
Supplement: Supplementary file 6 — CRF and ERF predictions for individual TmY4 and TmY9 cells. Analogous to Supplementary Data 3, but for TmY target types. Shown are the top four monosynaptic pathways, the strongest pathway passing through each of the top ten intermediary types (ranking from Extended Data Fig. 7), and the trisynaptic pathway Tm1–TmY–Dm3–TmY (see the section entitled Prediction of spatial normalization). [file 41586_2024_7953_MOESM6_ESM.zip › DataS4/TmY9qΓèÑ/720575940616936633.pdf]

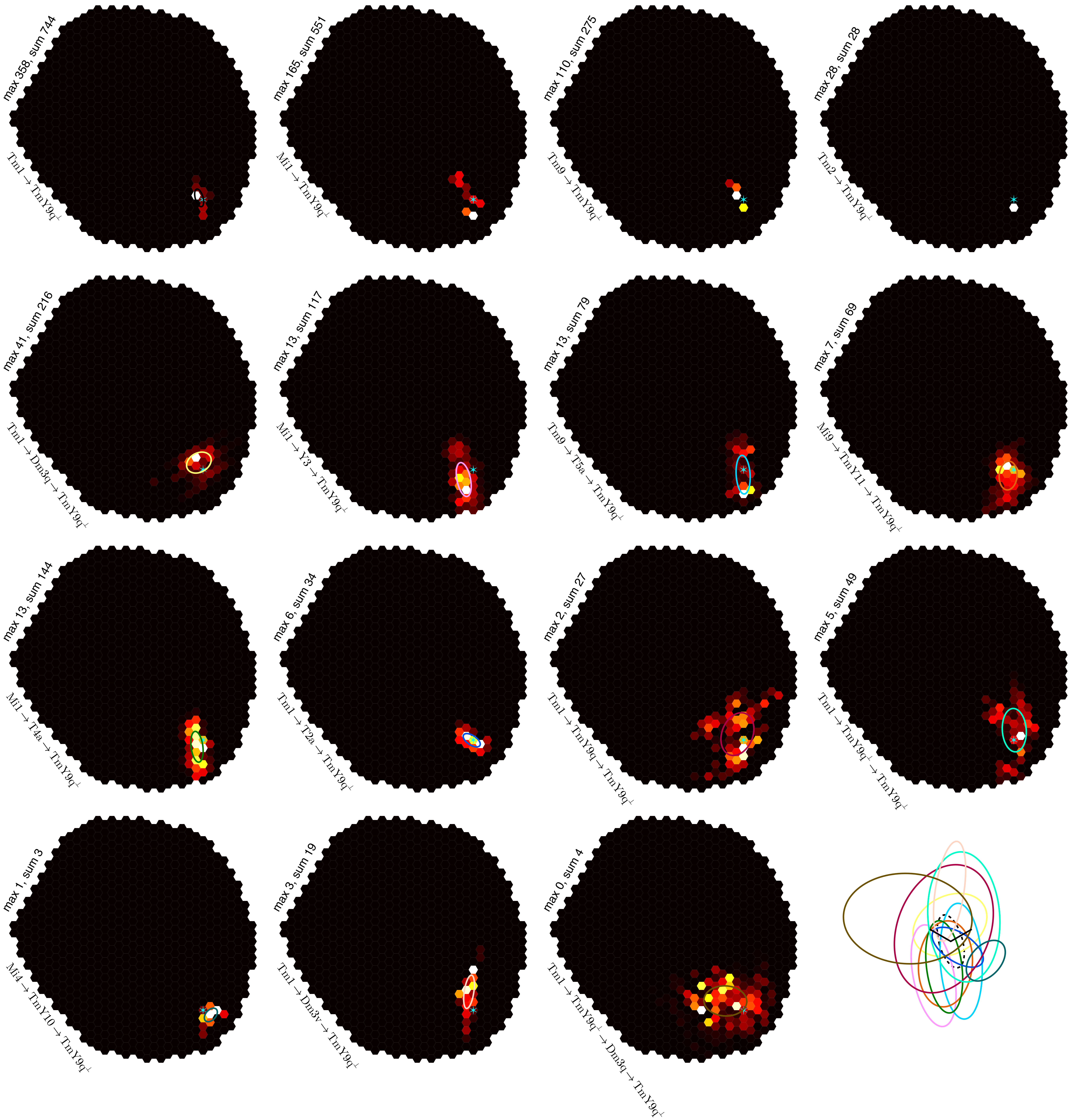
max 358, sum 744
Tm1 → TmY9q⊥
max 165, sum 551
Mi1 → TmY9q⊥
max 110, sum 275
Tm9 → TmY9q⊥
max 28, sum 28
Tm2 → TmY9q⊥
max 41, sum 216
Tm1 → Dm3q → TmY9q⊥
max 13, sum 117
Mi1 → Y3 → TmY9q⊥
max 13, sum 79
Tm9 → T5a → TmY9q⊥
max 7, sum 69
Mi9 → TmY11 → TmY9q⊥
max 13, sum 144
Mi1 → T4a → TmY9q⊥
max 6, sum 34
Tm1 → T2a → TmY9q⊥
max 2, sum 27
Tm1 → TmY9q → TmY9q⊥
max 5, sum 49
Tm1 → TmY9q⊥ → TmY9q⊥
max 1, sum 3
Mi4 → TmY10 → TmY9q⊥
max 3, sum 19
Tm1 → Dm3v → TmY9q⊥
max 0, sum 4
Tm1 → TmY9q⊥ → Dm3q → TmY9q⊥
